# Supplementary material for: Complex‐centric proteome profiling by SEC‐SWATH‐MS
Source: Mol Syst Biol. 2019 Jan 14;15(1):e8438. doi: 10.15252/msb.20188438 (PMC6346213; doi:10.15252/msb.20188438)
Supplement: Supplementary file 8 — Dataset EV7 [file MSB-15-e8438-s008.zip › feature_plots_string/P51610.pdf]

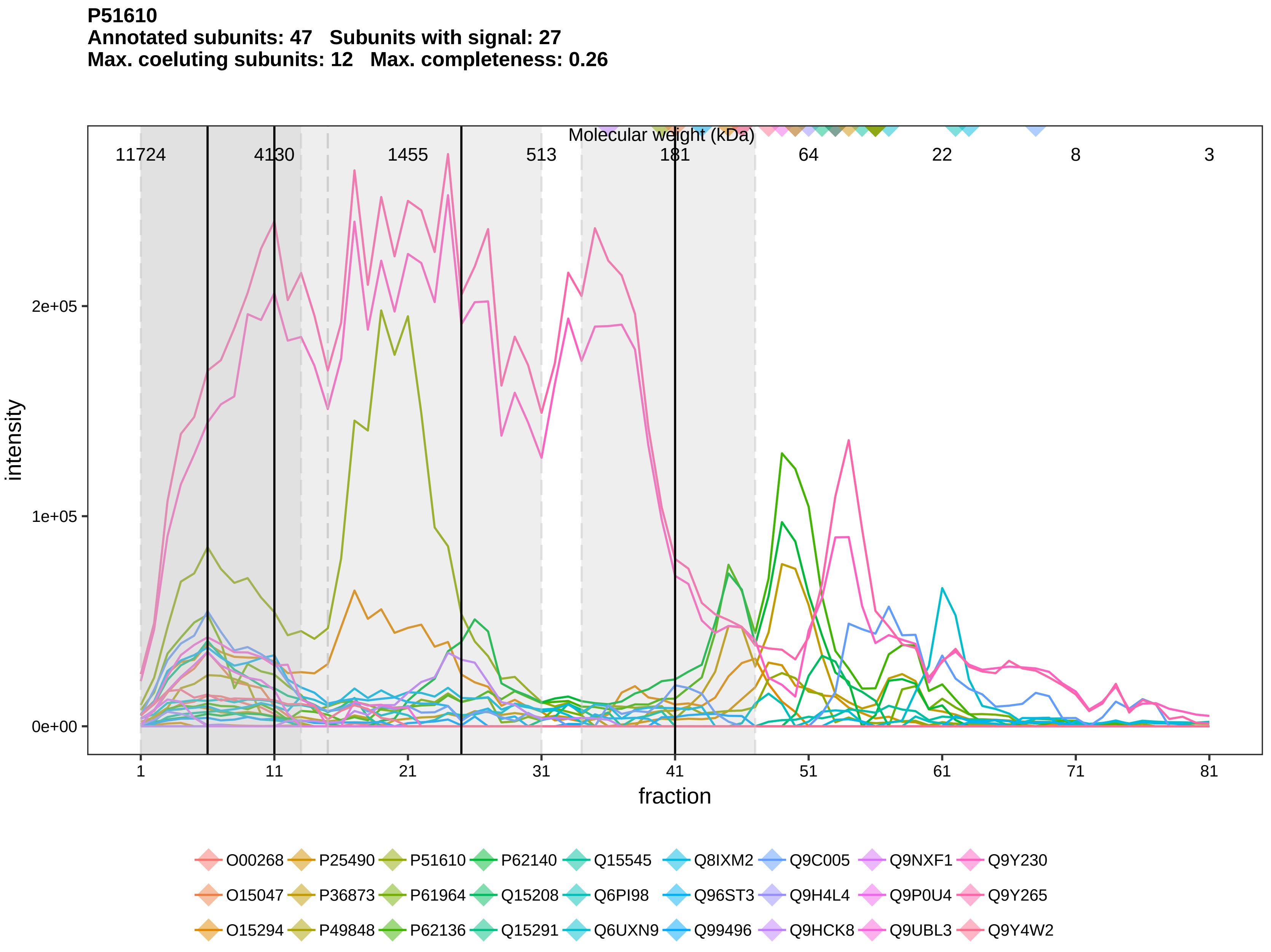

P51610
Annotated subunits: 47   Subunits with signal: 27
Max. coeluting subunits: 12   Max. completeness: 0.26
Molecular weight (kDa)
11724
4130
1455
513
181
64
22
8
3
intensity
2e+05
1e+05
0e+00
fraction
1
11
21
31
41
51
61
71
81
O00268
O15047
O15294
P25490
P36873
P49848
P51610
P61964
P62136
P62140
Q15208
Q15291
Q15545
Q6PI98
Q6UXN9
Q8IXM2
Q96ST3
Q99496
Q9C005
Q9H4L4
Q9HCK8
Q9NXF1
Q9P0U4
Q9UBL3
Q9Y230
Q9Y265
Q9Y4W2
